# What would Karl Popper say? Are current psychological theories of ADHD falsifiable?

**DOI:** 10.1186/1744-9081-5-15

**Published:** 2009-03-03

**Authors:** Katherine A Johnson, Jan R Wiersema, Jonna Kuntsi

**Affiliations:** 1 School of Psychology and Trinity College Institute of Neuroscience, Trinity College Dublin, Dublin 2, Ireland; 2 Department of Experimental-Clinical and Health Psychology, Gent University, H. Dunantlaan 2, B-9000 Gent, Belgium; 3 MRC Social, Genetic and Developmental Psychiatry Centre, Institute of Psychiatry, King's College London, De Crespigny Park, London, SE5 8AF, UK

## Abstract

Attention Deficit Hyperactivity Disorder (ADHD) is a common and highly heritable neurodevelopmental psychiatric disorder. Here, we critically review four major psychological theories of ADHD – the Executive Dysfunction, the State Regulation, the Delay Aversion and the Dynamic Developmental – on their abilities to explain all the symptoms of ADHD, their testability and their openness to falsification. We conclude that theoreticians should focus, to a greater extent than currently practiced, on developing refutable theories of ADHD.

## Background

Attention Deficit Hyperactivity Disorder (ADHD) is a common neurodevelopmental psychiatric disorder [1]. Much of the research into ADHD has focused on two areas – genetics and the neuropsychological/behavioural symptoms of the disorder. ADHD is one of the most heritable psychiatric disorders, with estimates of heritability at approximately 76% [2]. Although a number of genes have been associated with ADHD, the small odds ratios for these associations suggest that many genes may be involved and that these genes may each have a small effect [2,3]. Research is also continuing into the role of environmental factors and their interactions with genes and epigenetic processes in the development of the symptoms of ADHD [4,5]. The diagnosis of ADHD is based on the grouping of symptom presentation (impulsivity-hyperactivity and inattentiveness) and allows for three subtypes – impulsive-hyperactive, inattentive and combined-type [1]. These subtypes may have different aetiologies and neuropsychological/behavioural profiles. Psychologists and psychiatrists have developed a range of theories to explain the behaviour of children and adults with ADHD. It is argued here that any major psychological theory of ADHD must be able to explain these diagnostic symptoms. This paper aims to (1) provide a philosophical context for scientific theory-making and (2) critically review the four major theories of ADHD and consider the extent to which the predictions of each theory are testable and falsifiable.

## Philosophical context

According to the scientific philosophy of Popper, a scientific hypothesis must be testable and therefore open to falsification [6]. If a scientist can specify in advance an experiment that can falsify the hypothesis, then and only then is the hypothesis scientific. Popper argued that whilst it was easy to obtain confirmations or verifications for nearly every theory when one looked for a confirmation, a 'good' scientific theory forbade certain things to occur. A genuine test of a theory should lead to an attempt to refute it [7]. Popper suggested Einstein's theory of gravitation as an example of a theory that clearly satisfied the criterion of falsifiability. In contrast, he proposed that the two psycho-analytic theories of Freud and Adler were examples of theories that were non-testable and irrefutable (p.37) [7]. As Chalmers writes, "Usually, potential falsifiers will involve the specification of an experimental set-up designed to test a theory together with the description of an outcome inconsistent with the prediction of the theory" (p.68) [8]. If a current theory is proved false, then new theories will be developed that take into account the discriminating case. For a hypothesis to be testable, it must be well-described and precise, so as to allow for falsification and replication [6].

Other philosophers have argued that in practice, when anomalous (non-predicted) results are reported and accepted as real data, rather than abandoning that theory, scientists are more likely to modify their existing hypotheses to align with the new findings (or alternatively ignore the new results) [9]. According to Lakatos, a scientific theory (or "research program") has a "hard core", a hypothesis central to that theory. The hard core is stubbornly defended against criticism and refutation by "auxiliary hypotheses", produced in the light of new findings. Research programs fall into two types: progressive and degenerative. A degenerative program is marked by a lack of new facts and by lack of growth. In comparison, the progressive program generates new facts, new hypotheses and is the subject of growth. Scientific progression occurs when the auxiliary hypotheses of the progressive program aid the discovery of new knowledge, whilst the degenerative program is abandoned [9]. It has also been argued that new scientific findings improve the current set of hypotheses until there comes a point in time where a fresh and new hypothesis comes into being that starts a paradigm shift in thinking about that scientific topic [10].

The strength and health of the field of ADHD behavioural research (and ADHD genetic research, as a concomitant), is dependent on proposed hypotheses being made testable and falsifiable. ADHD is currently a behavioural construct, defined by behavioural dysfunction rather than by a biological marker [11]. It is a complex, multifactorial disease entity and as such, it is important that testable hypotheses of dysfunction are produced [12].

## Key behavioural theories of ADHD

This review focuses on four key cognitive and behavioural theories of dysfunction associated with ADHD: the Executive Dysfunction, Delay Aversion, State Regulation and the Dynamic Developmental theories. Each of these theories has undergone transformations over the last decade. As Lakatos predicted, theoreticians have added auxiliary hypotheses to explain new data as they have arisen; some of these auxiliary hypotheses are components of the other leading theories.

This review does not attempt to be systematic; rather it aims to review each of the theories from a philosophical perspective. After defining each theory, we asked these questions: (1) Does the theory explain all of the symptoms of ADHD? (2) Is the theory testable? (3) Has a falsifiable hypothesis been stated?

## The Executive Dysfunction theory of ADHD

### Definition

Executive dysfunction is a term used to explain deficits in "higher-order" cognitive processes, such as planning, sequencing, reasoning, holding attention to a task, working memory, inhibition of inappropriate and selection of appropriate behaviours [13-15]. These supervisory processes control, regulate and manage the "lower-level" cognitive operations, such as language, perception, explicit memory, learning and action. Executive functioning involves the operation of neural circuits that link the frontal cortices with the basal ganglia, thalamus and parietal cortices [16,17]. Anatomical and functional studies have found evidence of structural differences (e.g. [18]) and altered activation of the prefrontal cortex, fronto-parietal and fronto-striatal circuits in children with ADHD [19-23]. Dopaminergic and noradrenergic neurotransmitter dysfunction are implicated in the disorder [24]; these neurotransmitters are critical to the functioning of the fronto-striatal and fronto-parietal circuits. Although the use of these neural circuits in the theorisation of ADHD is not specific to the Executive Dysfunction hypothesis they are directly relevant to this hypothesis.

The Executive Dysfunction theory of ADHD suggests that the symptoms of ADHD arise wholly as a result of a reduction in executive control, which is caused by abnormalities in the structure, function and biochemical operation of the fronto-parietal and fronto-striatal neural networks (for a recent meta-analysis of executive function in ADHD, see [25]). Neuropsychological tests that are sensitive to the workings of the executive function system have been used to assess children with ADHD. The results of these tests have been directly (via fMRI and EEG) and indirectly (via behavioural studies) related back to the physiological, anatomical and biochemical dysfunctions within the frontal cortex, the fronto-parietal and fronto-striatal circuits in ADHD.

### Does the theory explain all of the symptoms of ADHD?

In terms of the three broad symptom types of ADHD, the Executive Dysfunction theory explains impulsivity and inattention, but has largely ignored the hyperactivity element of ADHD. Response inhibition, as the neuropsychological marker of impulsivity, has been earmarked by some as the critical deficit in ADHD, leading to secondary impairments in other executive functions, specifically working memory, internalisation of speech, self-regulation of affect-motivation-arousal and novel goal-directed action, resulting in decreased motor control of internally represented information and action [26]. Response inhibition has been investigated using neuropsychological tasks such as the go/no-go and stop signal in children and adults with ADHD (see below in Delay Aversion section for further discussion on the stop signal task). Different forms of attentional dysfunction in ADHD have also been investigated, with many studies using forms of the continuous performance task (CPT) to assay sustained attention deficits in children [27-30] and adults [29,31] with ADHD, and have related the findings back to deficits in the executive functioning circuitry. Posner's influential theory of attention [32] has been used to parse the roles of the proposed alerting, orienting and executive control attention networks, with evidence of dysfunction in the alerting and executive control networks in children with ADHD [33]. Within this framework, predictive value of the Executive Dysfunction theory is highlighted, as this theory predicts and presents evidence of dysfunction in other executive functions in ADHD apart from the three broad symptom types that define this disorder. This assertion is supported by evidence from neuropsychological studies investigating higher-order cognitive processes such as working memory [34,35], planning [36,37] and temporal processing [38,39].

Whilst there is supporting evidence of deficits in performance by participants with ADHD on executive functions such as response inhibition, sustained attention and spatial working memory, the recent Willcutt *et al*., meta-analysis concluded that the Executive Dysfunction theory failed to explain the full complexity of symptoms of ADHD [25]. The weighted mean effect size for all 13 executive function measures evaluated was .54, with the range (d = .43 – .69) considered to be of *medium *effect size. Executive dysfunction is not always found in all children with ADHD. For example, in a recent study, an estimated 35–50% of ADHD combined type cases showed response inhibition deficits [40]; yet we also note that a division of cases into those who show deficits and those who do not is essentially based on an arbitrary cut-off point on a continuous dimension.

Neuropsychological tests of executive function are often quite complex and involve a number of different executive functions, making it difficult to resolve the exact locus of dysfunction [25]. Additionally, the root cause of poor performance on the neuropsychological tasks might lie with a motivational or a state regulation deficit that causes a down-regulation of the neural circuits associated with executive functioning. Therefore, the Executive Dysfunction theory cannot be said to explain all of the symptoms of ADHD.

### Is the theory testable?

Many papers have used the Executive Dysfunction theory to produce testable hypotheses about the role of the frontal cortices and their circuits in the symptomatology of ADHD. One difficulty, however, with the Executive Dysfunction theory is the complexity in defining and then testing an executive function. The behavioural manifestations of ADHD probably represent a combination of cognitive and motor processes. For example, response inhibition, as measured by the Stop Signal task, is the summation of cognitive processes (such as sustained attention, goal-orientation and target detection) and control of the primed motor response [41,42]. Ideally, the contribution of each of these processes would be taken into account when formulating hypotheses for testing the response inhibition capacities of participants. The potentially poor understanding of the contribution of each of these processes may be one explanation for the inconsistency in the results from behavioural and functional imaging studies for some of the executive functions studied.

### Has a falsifiable hypothesis been stated?

In its most general form, the Executive Dysfunction hypothesis in ADHD is difficult to falsify, as poor performance on a general executive function task is taken as evidence of an executive function deficit in ADHD, without conditions specified for testing alternative hypotheses. This need not to be the case however and studies have been carried out where the cognitive process under study has been precisely identified (using careful control conditions) and the predictions for an alternative have been explicitly stated and tested (e.g. [43]). This raises the possibility that while ADHD is associated with poor performance on aspects of executive functioning, this poor performance may not be limited to executive dysfunction per se but may be part of a more general deficit or process [44]. For future progress, we propose that future studies on aspects of executive functioning in ADHD aim to state explicitly a falsifiable hypothesis.

## The State Regulation Model

### Definition

The State Regulation hypothesis states that a non-optimal energetic state could explain performance deficits in children with ADHD. This hypothesis is based on research using the Cognitive Energetic model of Sanders [45,46]. In this model, the efficiency with which a task is performed is considered to be a product of elementary cognitive stages and their energy distribution. The elementary stages are stimulus encoding, memory search, binary decision and motor preparation [47] and may be seen as structural computational information processes. The availability of these processes is related to the arousal and activation levels of the subject. Arousal is defined as a time-locked phasic physiological response to input, whereas activation refers to a long-lasting voluntary readiness for action [48,49]. Effort is necessary to meet task demands and to compensate for a sub-optimal state of arousal and/or activation by either activating or inhibiting the arousal and activation levels. The effort system is under control of an evaluation mechanism, which scans the momentary state of the arousal and activation levels. The state regulation theory states that children with ADHD have difficulty in keeping an optimal activation state, possibly due to inefficient extra effort allocation. Using Sternberg's additive factor method (1969), Sergeant and van der Meere (for reviews, see [50-53]) found the encoding, memory search, and decision stages to be intact. However, deficient response organization was noted, especially when stimuli were presented slowly. Later studies also noted that children with ADHD tend to perform more poorly in conditions of relatively slow, compared with fast and moderate event rates. The typically slow and variable response style in ADHD, when stimuli are presented slowly, is a consistent finding in these studies, whereas with respect to errors of commission, findings are mixed [54-57]. The robustness of the response time (RT) event rate effect also remains under sustained attention conditions of more than 30 minutes. Children with ADHD were found to have a rapid decline in task efficiency over time with a slow presentation rate, but not with a fast presentation rate (for a review, see [52]). According to the Cognitive Energetic model, event rate influences the motor activation level. Activation levels increase with an increase in event rate, whereas slow event rates may induce under-activation. To compensate for a sub-optimal activation state, extra effort allocation is necessary. Consequently, the event rate RT findings may suggest that children with ADHD are easily under-activated and have difficulty in adjusting their under-activated state because of insufficient extra effort allocation. Effort allocation has its physiological costs; hence further testing of the state regulation hypothesis may be critically dependent on the development of direct measures of the energetic pools [50]. In this vein, psychophysiological studies have been recently carried out. Children with ADHD showed higher heart rate variability (HRV) in the slow condition only, suggesting less effort allocation [58]. Using the event-related potential (ERP) methodology, Wiersema and colleagues showed that the poor performance of children with ADHD in the slow condition was related to a missing increase of the parietal P3 amplitude [59], which may be an indicator of effort allocation [60,61]. The same results were found for male adults with ADHD, indicating that problems in state regulation may persist in adult ADHD [62]. In conclusion, several studies indicate that event rate, which has been argued to have its locus in the activation pool, plays an important role in task performance in ADHD. Recent psychophysiological studies underscore the hypothesis of a state regulation deficit in ADHD and highlight the disturbed involvement of the effort pool in ADHD, especially in relation to an under-activated state. Another factor argued to influence energetic state and to optimise performance of children with ADHD is motivation. As Luman et al. (2005) have noted [63], there is clear evidence that motivational factors such as reward and response cost have a positive effect on performance of both typically developing children and children with ADHD. In some studies, however, reward was more beneficial for children with ADHD than for controls [64,65]. In a recent study, both factors (event rate and incentive) were combined and ADHD was associated with greater improvement in RT variability from baseline to fast-incentive condition [66]. According to the Cognitive Energetic model, effort allocation and motivation are strongly related. Hence, the sensitivity for reinforcement contingencies in children with ADHD would be interpreted, in state regulation terms, as evidence for a lack of effort allocation in ADHD.

Several issues regarding the State Regulation hypothesis remain. Direct supportive evidence for disturbances in the activation pool in ADHD is limited. Only a few studies have tried to directly measure the motor activation pool. Besides the cardiac response studies of Börger and colleagues [58,67], most of the evidence for disturbed motor activation comes from studies reporting Contingent Negative Variation (CNV) differences [68-70]. Effects in the first CNV (orienting) wave, however, have been reported more often than in the late CNV (motor readiness) and these studies did not include an event rate manipulation. Moreover, as the state regulation model is based on research using the cognitive-energetic model, a distinction is made between arousal and activation. Yet psychophysiological evidence for this distinction is limited and more research is warranted. The higher theta/beta ratio in the EEG signal, often found in ADHD populations (see for review [71]), has been argued by most investigators to be an indication of cortical under-arousal.

Although most evidence suggest that the state regulation problems in children with ADHD are related to under-activation problems, originally it was suggested that activation and performance take an inverted 'U' function where either increases or decreases in activation from an optimal energetic state lead to performance decrements [72]. In order to test whether ADHD is also related to over-activation, more than two conditions of presentation rate should be used: not many studies have done this. Nevertheless, there is some data supporting the inverted 'U' predictions. Children with ADHD were found to have problems with response inhibition in a fast and slow condition, but performed equally well as controls in a medium condition [72]. Sonuga-Barke (2002) [73] found children with ADHD to experience the largest problems with time use on trials with a short and long duration, while they performed equally well in trials with a medium duration. Finally, it is not clear which exact brain areas underlie the state regulation problems in ADHD. Although several brain structures and neurotransmitters have been argued to be associated with the different energetic mechanisms [48,49] few attempts have been made to investigate this directly.

### Does the theory explain all of the symptoms of ADHD?

The state regulation account argues that ADHD symptoms may increase or decrease depending on the child with ADHD's state. For example, symptoms of inattention may appear when tasks are slow or boring. Children may become impulsive or hyperactive in an attempt to increase stimulation (self-stimulation). This may explain findings such as longer RTs, higher intra-subject variability of responding and increased error rates in children with ADHD.

With respect to the specificity of a state regulation deficit in ADHD, the following findings should be emphasised. Performance in adults with High Functioning Autism (HFA) was disproportionally impaired by a fast event rate condition, whereas no difference between groups was noted in a slow condition [74]. Poor RT performance occurred independently of event rate in children with ADHD and comorbid Tic Disorder [72], in children with early- and continuously treated phenylketonuria (PKU) [75,76], in learning disabled children without ADHD [57], and in children with Mild Mental Retardation plus Conduct Disorder [77].

### Is the theory testable?

Crucially, the state regulation account suggests that differences between children with ADHD and typically developing children will be minimal when children with ADHD are in an optimal state. Unfortunately, it is difficult in practice to specify the optimal state as this may be task/context dependent and will also differ between children.

### Has a falsifiable hypothesis been stated?

Falsifiable hypotheses have been stated. The specificity of these hypotheses, however, is not always clear. Most evidence suggests that the state regulation problems in children with ADHD are related to under-activation problems, however originally it was suggested that either increases or decreases in activation from an optimal energetic state lead to performance decrements in ADHD [72]. One way to improve the testability of this hypothesised inverted U function, is to incorporate more than two event rates, including an individually-based optimal event rate.

## The Delay Aversion and Dual Pathway theories

### Definition

The Delay Aversion theory was first described by Sonuga-Barke and colleagues in the early 1990s [78,79] and has undergone a recent elaboration process to incorporate elements of the Executive Dysfunction theory [80,81], providing an excellent example of Lakatos' auxiliary hypothesis. The Delay Aversion hypothesis accounted for the finding that children with ADHD symptoms 'can wait but often don't want to'. The original delay aversion hypothesis predicted that children with ADHD are not impulsive in the sense of always opting for an immediate reward at the expense of overall rewards, but that they do so only in circumstances where this leads to a shorter overall delay. It is a motivational account of ADHD, in contrast to theories focusing on cognitive deficits.

Inattentiveness and hyperactivity are considered to reflect attempts to reduce subjective experience of delay in situations where delay cannot be avoided [79]. The original paradigm's test of the hypothesis included two conditions, in each of which children made a choice, a fixed number of times, between a small immediate reward and a large delayed reward. In the 'no post-reward delay' (experimental) condition, choosing either an immediate small reward or a delayed larger reward led immediately to the next trial. In the 'post-reward delay' (control) condition, choosing the small immediate reward led to an extra delay period so that the length of each trial was equated and independent of the choice made. A group difference was found only in the 'no post-reward delay' condition, with children with ADHD symptoms choosing the small, immediate reward more often than control children [78]. The finding of an absence of group difference in the control condition was replicated with preschool children [82]; but see [83].

More recently, Sonuga-Barke incorporated the delay aversion hypothesis within a new framework, the Dual Pathway theory [80,84]. This theory proposed the existence of two distinct subtypes ('pathways') within combined type ADHD: one characterised by inhibitory deficits and the other by delay aversion. The theory predicted that the pathway involving inhibitory deficits is linked to the meso-cortical dopamine branch, is categorical in nature (i.e. children with ADHD are qualitatively different from other children) and less strongly associated with genetic factors than the second pathway. In contrast, the pathway involving delay aversion is linked to the meso-limbic dopamine branch and 'disturbances in reward centers', and is proposed to reflect a continuously distributed trait that is under stronger genetic influence. Further elaboration on the model [80] included consideration of additional factors, such as compensatory strategies, that may contribute to task performance in children with ADHD.

Sonuga-Barke (2002) [84] developed the dual pathway model based on data from a study that compared performance of children with ADHD and comparison children on an inhibition task (stop task) and a delay aversion task (choice-delay task) [85]. Whereas poor performance on both tasks was associated with ADHD, there was a lack of a significant association between inhibition and delay aversion performance, leading to the argument of two independent pathways. Sonuga-Barke and colleagues have since reported a similar lack of an association between inhibition [82] or 'executive function' [86] and delay aversion performance also in younger (pre-school) children.

Recently the concept of delay aversion has also been revised such that it no longer is viewed as a competing causal model with impulsivity-as-lack-of-self-control [87,88]. Delay aversion is viewed as contributing to choice impulsivity, observed on the choice-delay paradigm as a relative increase in choice impulsivity in the no post-reward delay condition [88]. Hence, whereas the original formulation predicted a significant ADHD-control group difference only in the no post-reward delay condition, the revised formulation predicts group differences under both conditions (choice impulsivity), although a larger effect for the no post-reward delay condition (delay aversion specific effect). The new data from a large international sample support the revised formulation of the theory [88].

### Does the theory explain all of the symptoms of ADHD?

The main focus of the theory is on impulsiveness. Inattentiveness and hyperactivity are considered to reflect attempts to reduce subjective experience of delay in situations where delay cannot be avoided [79].

### Is the theory testable?

The original delay aversion hypothesis that contrasted the different conditions [78] included testable hypotheses. The revised formulation (e.g. [88]) includes the related testable hypothesis of a statistical interaction between diagnostic group and delay condition. The more general prediction of an association between ADHD and choice impulsivity is not specific to delay aversion theory but is shared for example with the Dynamic Developmental Theory of Sagvolden and colleagues (see below).

The dual hypothesis includes several predictions, including that of the two subtypes, but to our knowledge this is yet to be investigated at the level of individual children. The dual pathway model relies on a correlational pattern of findings, the interpretation of which is somewhat difficult at present. The effects of combining ADHD and control groups in the analyses are unknown. Further development of the model would benefit from a clear description of tasks and variables that measure the proposed constructs. Many of the links proposed in the model are yet to be tested and, overall, replication and extension of findings with independent samples will be important. There are no studies, as yet, describing the neural pathways or role of genetics in delay aversion behaviour in ADHD [89].

Beyond the proposal of the two subtypes, the dual pathway model includes the assumption of poor performance on the Stop Task as reflecting an inhibition deficit. There is ERP evidence to suggest that slower SSRT may be related to early problems with shifting attention to the stop signal, questioning the validity of the SSRT as an inhibition measure [90]. Meta-analyses of the SSRT in ADHD have also concluded that SSRT differences between children with and without ADHD do not reflect real differences in stopping speed (inhibition) but reflect differences in mean reaction time to go stimuli [91]. Another issue regarding the Stop Task concerns the difficulty of the task itself. In some studies, data from a large number of participants have been excluded due to concerns regarding the ability of the children to perform the task. For example, one study excluded Stop Task data from 27% of the participants due to very high omission and commission error rates (i.e. the worst performing children were excluded from the analysis), the effects of which are unknown [85].

### Has a falsifiable hypothesis been stated?

The original delay aversion hypothesis [78] included the falsifiable hypothesis of an ADHD-control difference in the no post-reward delay condition and its absence in the post-reward delay condition (and therefore an implicit group-by-condition interaction). The most recent formulation of the delay aversion theory [88] includes the prediction of a group-by-condition interaction effect. The Dual Pathway model was the first model to incorporate two theories of ADHD as an explanation for the many observations in ADHD. Yet more specific, testable hypotheses are required regarding performance on tasks and the proposed links within the model. Currently it is not clear which findings would specifically falsify or support the model.

## The Dynamic Developmental Theory of ADHD

### Definition

The Dynamic Developmental Theory (DDT) of ADHD has been developed by Sagvolden and colleagues over the past 20 years and has been the subject of a recent major review process [11]. This comprehensive theory attempts to explain the behavioural manifestations of ADHD from a neurotransmitter through to a societal level and aims to explain all symptoms of ADHD. Much of the data supporting this theory is based on animal data [92] and the theoretical underpinning of this theory is behaviourism. The theory suggests that there are two main behavioural mechanisms underpinning many of the symptoms of ADHD: altered reinforcement of novel behaviour and deficient extinction of inadequate behaviour [11]. The basis for this theory lies in the delay-of-reinforcement gradient between a response to a stimulus and a reinforcement of that response [93]. The efficacy of the reinforcer is greater if the delay between the response and the reinforcement is smaller rather than larger. It is hypothesised that in ADHD, the critical "window of opportunity" for the reinforcer to take effect is smaller than for normal children. The result is that socially desirable behaviour is not reinforced in time, leading to many of the symptoms of ADHD. Extinction occurs when delivery of the reinforcer stops and subsequently the response is not elicited. Extinction may occur in time with a phasic decrease in tonic levels of dopamine. It is hypothesised that in ADHD the extinction process will be faulty because of the lowered tonic level of dopamine [11].

The DDT has adapted some of the findings from the Executive Dysfunction and Delay Aversion literature, incorporating attentional, behavioural organisation, motor coordination, nondeclarative habit learning deficits and delay aversion, as auxiliary hypotheses, into a larger dopaminergic, fronto-striatal neurological model. Reinforcement and extinction processes are hypothesised to be the core problems in ADHD due to abnormally low levels of dopamine, affecting the functioning of the anterior cingulate, dorsolateral prefrontal and motor circuits and subsequently a variety of behaviours.

### Does the theory explain all of the symptoms of ADHD?

The DDT holds a theoretical position on hyperactivity, impulsivity and inattention. The shorter delay-of-reinforcement gradient and deficient extinction effect in ADHD is hypothesised to occur within the mesolimbic dopamine branch along the anterior cingulate-fronto-striatal circuit. Hyperactivity, impulsivity and delay aversion are explained through this system. Hyperactivity may be due to a combination of factors including failing extinction resulting in too many responses, a deficit in the pruning of ineffective or inappropriate responses resulting in a relative increase in these, and a short delay-of-reinforcement gradient resulting in poor reinforcement of appropriate behaviour. Impulsiveness may be due to the short delay-of-reinforcement gradient, as the appropriate behaviour is not reinforced well and the significance of the immediate reinforcer is much stronger then the delayed reinforcer, which remains unlinked to the original response. Delay aversion is also explained by the shortened delay-of-reinforcement gradient. Stronger and more salient reinforcers are required to produce the desired behaviour in children with ADHD, compared with control children. The larger but delayed reward loses its saliency [11].

Abnormally low dopamine levels within the mesocortical and nigrostriatal branches are hypothetically linked with poor reinforcement and extinction processes in the dorsolateral prefrontal and motor circuits, respectively. This is hypothesised to lead to deficient attention and poor behavioural organisation (dorsolateral prefrontal circuit) and poor motor coordination, response disinhibition and nondeclarative habit learning (motor circuit) [11].

Behavioural variability may be due to a combination of an increased number of types of responses and a deficient extinction process resulting in a greater number of inappropriate responses. Variation between people with and without ADHD may be due to variation within the biological starting position of the person (genes and pre-natal environment), the delay gradient and the ongoing neuromodulatory effects of the environment (learning and experience).

### Is the theory testable?

The DDT is testable. The altered-reinforcement hypothesis makes two explicit predictions [94]. (1) The delay-of-reinforcement gradient is steeper for children with ADHD than controls, meaning that the retroactive effect of a reinforcer is shorter with children with ADHD [95]. A reinforcer in close proximity to a response will be more effective for these children. High-frequency responding (hyperactivity) and fast responses (impulsiveness) should manifest as a function of the number of reinforcers delivered, rather than present at the beginning of a task. (2) If there is a short delay gradient in ADHD, then there will be a weakening in the association between the response and the reinforcer, thus negatively affecting the percent correct responses (sustained attention). These predictions were supported through testing of spontaneously hypertensive rats (SHR), a putative animal model of ADHD [96] and children with and without ADHD [94,97,98].

Researchers may interpret the results as suggestive of deficits in spatial working memory and indeed Aase and Sagvolden suggest that reduced working memory capacity may be underlying the performance of the children with ADHD [99]. The rate of reinforcer presentation is also of importance and reminds one of the theoretical positions of the State Regulation hypothesis. When the rate of reinforcement was low (and infrequent), children with ADHD showed deficits in the number of correct responses and variability in response on the task, but normal performance on the frequency of responding and the number of very short responses. When the rate of reinforcement was high (and thus often), then no performance decrements were found in the ADHD group, particularly in the younger children [97].

### Has a falsifiable hypothesis been stated?

The DDT provides a well described theoretical framework that has produced falsifiable hypotheses within the confines of the stimulus-response-reinforcer experimental set-up. Theoreticians of the DDT relate the theory to everyday behavioural manifestations of ADHD [100], for example in terms of sustaining attention and learning.

## Conclusion

In this review we have considered the strengths and weaknesses of the four major psychological theories of ADHD, especially as they relate to the requirement for scientific hypotheses to be open to falsification [6]. We conclude that hypotheses relating to specific aspects of executive functioning in ADHD have the potential to be falsifiable; yet, in most published studies to date this requirement has not been fulfilled. The state regulation account proposes hypotheses that are falsifiable in principle, but in practice in studies using cognitive-experimental tasks an optimal state may be difficult to induce in a laboratory setting. The most recent formulation of the delay aversion theory includes the falsifiable hypothesis of an interaction effect between diagnostic group and delay condition, but also includes components that are not specific to the delay aversion hypothesis. The dual pathway model requires further refinement and for falsifiable hypotheses to be made explicit. The DDT is grounded in a well articulated, scientific framework but needs to be extended further into the human experimental setting.

Reflecting on these four theories of ADHD, it is striking that the researchers coming from different theoretical positions may be describing the same phenomena but utilising different words, concepts and schemas; they may also be defining the same phenomena or process but from a different temporal or anatomical point of view. For example, an arousal deficit in ADHD (lower energetic pool) may affect motivation to complete a task (poor state regulation) or the ability to sustain attention to a task (executive dysfunction), which behaviourally may manifest as an aversion to delay and poor performance on some executive function tasks; this behaviour may be reinforced inappropriately and because of low dopamine tone there may be deficient extinction of the socially inappropriate behaviour.

One common assumption of all the hypotheses is that there is a certain degree of homogeneity within the ADHD construct. It is possible that through a direct analysis of the models and an evaluation of how they dissociate and overlap, there will be a subgroup within the broader ADHD group that will show impairments on some other psychological aspect not currently incorporated within these four models. It is hoped that in this event, a new scientific model will be generated. In a similar manner, a comparison of children with ADHD with children with other clinical diagnoses may also inform theorisation.

Each of these theories is able, *post hoc*, to explain many of the phenomena we observe in ADHD; yet this is not where their strength lies and we need to move from post hoc theorisation further towards explicit, a priori (see also [25]) falsifiable and testable hypotheses. Whereas a synthesis of aspects of the different theories is one possibility, before we accept any single hypothesis that would form an aspect of this, each hypothesis needs to be precisely tested, step by step. To quote Popper, "The scientific tradition is distinguished from the pre-scientific tradition in having two layers. Like the latter, it passes on its theories; but it also passes on a critical attitude towards them. The theories are passed on, not as dogmas, but rather with the challenge to discuss them and improve upon them." (p.50) [7].

## Competing interests

The authors declare that they have no competing interests.

## Authors' contributions

KJ, RW and JK conceived the review idea, prepared, reviewed and approved the final manuscript.
